# Changes in Local School Policies and Practices in Washington State After an Unfunded Physical Activity and Nutrition Mandate

**Published:** 2011-10-15

**Authors:** Myde Boles, Julia A. Dilley, Clyde Dent, Miriam R. Elman, Susan C. Duncan, Donna B. Johnson

**Affiliations:** Program Design and Evaluation Services, Multnomah County Health Department and Oregon Public Health Division; Multnomah County Health Department and Oregon Public Health Division, Portland, Oregon; Multnomah County Health Department and Oregon Public Health Division, Portland, Oregon; Oregon State University and Oregon Health and Science University, Portland, Oregon; Oregon Research Institute, Eugene, Oregon; University of Washington, Seattle, Washington

## Abstract

**Introduction:**

Policies and practices in schools may create environments that encourage and reinforce healthy behaviors and are thus a means for stemming the rising rates of childhood obesity. We assessed the effect of a 2005 statewide school physical activity and nutrition mandate on policies and practices in middle and high schools in Washington State.

**Methods:**

We used 2002, 2004, and 2006 statewide School Health Profiles survey data from Washington, with Oregon as a comparison group, to create longitudinal linear regression models to describe changes in relevant school policies after the Washington statewide mandate. Policy area composite measures were generated by principal component factor analysis from survey questions about multiple binary measure policy and practice.

**Results:**

Relative to expected trends without the mandate, we found significant percentage-point increases in various policies, including restricted access to competitive foods in middle and high schools (increased by 18.8-20.0 percentage points); school food practices (increased by 10.4 percentage points in middle schools); and eliminating exemptions from physical education (PE) for sports (16.6 percentage-point increase for middle schools), exemptions from PE for community activities (12.8 and 14.4 percentage-point increases for middle and high schools, respectively) and exemptions from PE for academics (18.1 percentage-point increase for middle schools).

**Conclusion:**

Our results suggest that a statewide mandate had a modest effect on increasing physical activity and nutrition policies and practices in schools. Government policy is potentially an effective tool for addressing the childhood obesity epidemic through improvements in school physical activity and nutrition environments.

## Introduction

Since 1980, the prevalence of obesity among children and adolescents in the United States has tripled (1). From 1980 to 2008, the prevalence of obesity among children aged 6 to 11 years increased from 6.5% to 19.6%, and among adolescents aged 12 to 19 years obesity increased from 5.0% to 18.1% (2). Policies and practices in schools may create environments that encourage and reinforce healthy eating and regular physical activity and thus are promising means to stem the rising rates of childhood obesity (3,4).

To address the youth obesity problem, state and federal authorities have adopted obesity-prevention strategies such as legal mandates to improve school physical activity and nutrition policies. The federal Child Nutrition and WIC Reauthorization Act of 2004 mandated that all US school districts participating in the federally reimbursed school meal programs develop a local school wellness policy by the beginning of the 2006-2007 school year (5). Washington's legislature adopted Washington Senate Bill 5436, which was similar to the federal mandate and required each of Washington's 296 school districts to establish a nutrition and physical fitness policy by August 1, 2005, one year before the federal deadline. No funding was authorized for the implementation of SB 5436.

To assist school districts, the Washington State School Directors' Association developed a model policy regarding access to nutritious foods, opportunities for exercise, and classroom instruction related to nutrition and physical activity (6). The model policy required that middle school students have an average of 100 minutes per week (20 minutes per day) of aerobic education activity. High school students were required to complete 2 credits of health and fitness. It further recommended that districts adopt policies to hire certified physical education teachers; provide after-hours access to school facilities for physical activity, fitness, sports, and recreation programs; and identify safe routes to school for walking and biking.

For nutrition, the model policy required that school breakfasts and lunches meet the nutritional standards of state and federal school breakfast and lunch programs (7). In addition, the model policy contained provisions for the availability of fresh fruit and safe drinking water, use of nonfood alternatives for rewards, competitive pricing for healthy food options, adequate time and places to eat lunch, and a nutrition education curriculum focused on knowledge, skills, and assessment of personal eating habits.

Limited research has examined the relationship between state and federal legislation related to physical activity, nutrition, and school wellness and local physical activity, nutrition, and wellness policies. Research on the effect of the federal wellness policy mandate on local wellness policies in a nationally representative sample of schools in the 2006-2007 or 2007-2008 school years found that policies were weak overall and varied greatly from district to district (8). Among the few studies that specifically evaluated changes in local policies before and after the federal wellness mandate, 1 found that overall time available for physical activity did not change in a random sample of 45 rural elementary schools in Colorado after the policy went into effect (9). Another study of 847 medium and larger schools in the United States found that nutrition components increased significantly and were reported as the components most frequently implemented (10).

Previous studies examining the effect of a policy mandate on school policies have been limited by lack of both a comparison group and strong longitudinal data on policy trends. The primary objective of our study was to assess the effect of Washington's statewide mandate on physical activity and nutrition (PAN) policies and practices in schools relative to historical trends in Washington and to compare these with those in Oregon, a geographically and demographically similar state without a statewide PAN mandate. We hypothesized that an unfunded policy mandate may not lead to successful policy adoption and that its success depends on both components of PAN policies and characteristics of the school districts. Our secondary objective was to investigate whether the law's effect was associated with geographic area (urban or rural), school-level socioeconomic status (SES), or the average standardized test scores of schools. Washington and Oregon's demographic similarities make them ideal for examining how statewide laws lead to local policy changes.

## Methods

We used public health surveillance data to conduct a secondary data analysis. We performed hierarchical longitudinal linear regression with schools nested in time to test whether the proportion of schools in Washington with PAN policies and practices changed after the implementation of the statewide PAN mandate compared with schools in Oregon where there was no mandate.

### Data and sample

The School Health Profiles survey (Profiles) is conducted biennially by the Centers for Disease Control and Prevention (CDC) in collaboration with state and local education and health agencies. It is a self-administered survey of public secondary school principals and lead health education teachers and is designed to assess school health programs, policies, and activities (11). CDC uses a random, systematic, equal-probability sampling strategy to produce representative samples of public schools that serve students in grades 6 through 12. In 2004 and 2006, Washington modified this sampling procedure and invited all secondary schools, rather than just a sample, to participate. We obtained identical 2002, 2004, and 2006 data from principal and health educator surveys from the Oregon Public Health Division and the Washington State Department of Health. Study schools had an enrollment of at least 15 students per grade, had a standardized health and physical education curriculum, and were not alternative schools or combined middle/high schools (Table 1).

### Measures

We used the 30 nutrition-related questions and 26 physical activity-related questions in Profiles. Most item responses in Profiles were binary, indicating the presence or absence of a particular policy, practice, activity, or attribute at the school. We recoded items so that positive responses always indicated a better or desired condition, such as presence of a policy.

To create a manageable set of PAN outcome measures we performed a preliminary analysis of principal component factors to identify subsets of associated items within physical activity-related and nutrition-related domains. We began by grouping items in conceptual domains based on item content (eg, nutrition policy, nutrition curriculum). We then extracted the empirical principal components and used structural equation modeling to create item groupings  (12).

In the nutrition domain, the factor analysis procedure confirmed 7 independent sets of items — 3 factors relating to classroom educational content and 4 factors relating to nutritional policy. In the physical activity domain, we found 10 independent factors — 3 educational content factors and 7 policy-related factors (Appendix).

We combined items within a factor into composite outcome measures by summing. Measures with larger scores reflected a greater number of positive responses to component variables. Once combined, we recoded these measures to a percentage scale (ie, 0-100).

We used indicator variables for year, state, and introduction of the law (1 = presence of policy in Washington schools in year 2006, 0 = otherwise).

We considered schools with students in grades 6 to 8 as middle schools and schools with students in grades 9 through 12 as high schools. We examined middle and high schools separately because Washington's model policy gave different recommendations for each and because of other differences such as availability of competitive foods.

As school descriptors, we used publicly available school-level data from the 2 states' departments of education as covariates in the models. These data included SES (measured as the average percentage of students eligible for the free and reduced-price lunch program during study years; schools with more than one-third of students enrolled in the lunch program were considered low-SES schools); geographic area (urban and not urban using Rural Area Commuting Area codes; schools located in urban and suburban areas were classified as urban; schools located in small towns and frontier areas were classified as not urban) (13); and average percentage of students meeting state standards on statewide achievement tests (high performing schools were those with more than half of students meeting state standards).

### Data Analysis

To determine whether the Washington State mandate had an effect on the presence of PAN policies and practices in 2006, we created hierarchical longitudinal linear regression models with schools nested in time by using the GLMMIX procedure in SAS version 9.2 (SAS Institute, Inc, Cary, North Carolina). In the base model, we tested whether the proportion of schools in Washington reported having changed each of the 17 PAN policy and practice composite outcome measures from 2002 and 2004 (before the statewide mandate) to 2006 (after the statewide mandate). We used Oregon as an implicit control to strengthen these estimates. We included indicator variables "state," "year," and "law" in models for each PAN outcome and stratified by school type (middle school vs high school):

Base Model: policy_jst_ = β_0_ + β_1_state_s_ + β_2_year_t_ + β_3_law_st_ + ε_jst_

with *j* = 1 to number of schools;

*s* = 0 (Oregon), 1 (Washington); and

*t* = 0 (2002), 2 (2004), 4 (2006).

The coefficient β_0_ indicated the baseline (2002) percentage value of policy in Oregon; the coefficient β_1_ for the variable "state" measured the baseline difference between Washington and Oregon; the coefficient β_2_ for the variable "year" assessed secular annual trends across the study years; the coefficient β_3_ for the variable "law" determined the deviation from the expected trend in Washington in 2006; and ε indicated random error. We stratified the base models by grade and tested the model coefficients for consistency across school type using contrast statements in SAS.

To determine whether the Washington State mandate had similar effects across various school subgroups, we added terms to adjust for the covariates SES, geographic area, and school test score and incorporated terms for interactions of these variables with the enactment of the law.

We used a logistic link function in all models to test for statistical significance. Model coefficient estimates are presented in percentage-point scale values in our discussion and in Tables 3 through 5 in this article. We considered *P* values of less than or equal to .05 (2-sided test) as significant and estimates of 5 percentage points or more as substantively meaningful.

The Washington State and Oregon Public Health institutional review boards declared this study to be exempt under 45 CFR 46.101(b)(4).

## Results

Approximately half of all Washington State schools had high SES or high test scores (Table 2) At the 2002 baseline, only 31.6% to 41.6% of middle schools and 14.9% to 23.1% of high schools had policies in place that restricted access to competitive foods (what types of foods or times of day for access), and only 9.0% of middle schools and 17.7% of high schools had favorable school food practices (adequate time for lunch and availability of fruits and vegetables at school events) (Table 3).

In the absence of the Washington PAN mandate, the expected trends (based on 2002-2006 Oregon data and 2002-2004 Washington data) for the percentage of Washington schools with nutrition policies and practices generally did not change; however, the percentage of schools with restricted access to competitive foods (ie, what foods and when accessible) increased significantly, and healthy food options (ie, low-fat snacks, fruits, and vegetables) decreased significantly. The expected trends in physical activity policies and practices did not change significantly, except for a decline in the percentage of schools requiring certification for middle school physical education (PE) teachers.

Both middle schools and high schools showed a significant (18.8-20.0 percentage-point) increase in the number of schools with restricted access to type of competitive foods (Table 4). For restricted access to competitive foods (time of day), high schools increased by 19.2 percentage points, which is significantly higher than in middle schools  where the increase was not statistically significant. Unexpectedly, healthy food options for middle and high schools declined significantly, by 5.9 and 2.0 percentage points, respectively. Middle schools showed a significant (10.4 percentage-point) increase in school food practices (ie, adequate time for lunch and availability of fruits and vegetables at school events). There was no significant increase for high schools.

There was a significant increase in the percentage of middle schools that did not allow exemptions from PE for sports, community activities, or academics, and a significant increase in high schools that did not allow such exemptions for community activities (Table 5). Profiles found no other significant increases for other physical activity policies and practices.

When we examined the interactions of each of the 17 policy measures with urban/not urban, high/low SES, and high/low academic performance (middle and high schools combined), we found only 3 significant interactions: restricted access to competitive foods (type of food), which was present in 14.0% more higher-performing than lower-performing schools; restricted access to competitive foods (time of day), which was present in 11.3% fewer urban vs not-urban schools; and facilitators for PA (ie, safe routes to schools, community programs), which were available in 0.3% low- vs high-SES schools.

## Discussion

We found significant increases in the percentage of middle and high schools reporting the presence of certain PAN policies and practices after the implementation of the Washington State PAN mandate. This is the first published study to present a longitudinal analysis of changes in PAN policies and practices in 1 state by using measures of PAN policies and practices in another state for comparison to control for secular trend.

In 2002, few schools in Washington State had restricted access to competitive foods or nutrition-related policies in place, although most schools did have healthy food options available. It is not surprising that restricted access to competitive foods and school food practices were areas for growth, even in absence of the Washington mandate. We were surprised, however, to find a substantial decline in the percentage of middle and high schools offering healthy food options. Because the Profiles questions about healthy food options focused on the availability of healthy foods in vending machines and school stores, these schools may have been eliminating these venues for food purchases rather than reducing the availability of healthier food types in vending machines or school stores. Another explanation for the decline may be changing perceptions of school principals about what constitutes a "healthy" option.

Because so many Washington State schools already had physical activity policies and practices in place before the statewide mandate, opportunity for growth in this area was limited. The only area that changed after the implementation of the law was an increase in the number of schools that were not allowing student exemptions from PE because of participation in school sports and other school or community activities. Rather than trying to fund new provisions and programs for physical activity, the elimination of exemptions from PE may have been a budget-neutral way for schools and districts to respond to the mandate.

Our study's findings of a significant increase in nutrition policies and practices and only small improvement in physical activity policies are consistent with a recent study of trends in state-level school nutrition and physical activity policy environments (14). That study found that schools adopted more food service and nutrition policies than physical activity, education, or weight assessment policies. Similarly, a study of the effects of federal wellness legislation in school districts throughout the United States found that nutrition components were the most frequently implemented (10). However, even in the presence of nutrition policies, improvements in school food environments are modest (15), and foods of minimal nutritional value remain available (16). In contrast to most nutrition policies, many physical activity policies have a direct effect on instruction time (eg, requiring more PE classes or longer classes), and this may be a barrier to school districts adopting such changes.

We saw very few differences in change associated with various school-level factors, such as urban/not urban setting, SES, and academic performance. This suggests that the effect of a policy mandate was similar among different school types.

Washington State did not allocate funding to support schools in implementing their PAN policies, nor was there meaningful quality assurance of adopted policies or clear punitive measures in place for school districts that failed to effectively implement a policy. Addition of any of these supportive measures might have resulted in different — perhaps greater — policy and practice improvements.

This study has several limitations. First, we did not see appreciable increases in physical activity policies and practices associated with the Washington State mandate. This may indicate that Profiles did not ask about the features of the model policy that were most emphasized and acted on by local school districts. A substantial portion of the language in the model policy described the amount of instructional time for physical education, which was not asked about in the Profiles questionnaire. Second, we examined the changes in school policies and practices only 1 year after the Washington statewide PAN mandate went into effect. Arguably, 1 year is too short a time for schools to mobilize their efforts for PAN-related policy changes. However, we did see changes in restricted access to competitive foods, nutrition policy, and reduced exemptions from PE relative to trend, which could confirm that PAN effected changes. Conversely, a federal wellness policy requirement similar to the Washington policy requirement was scheduled for implementation in both Washington and Oregon in the fall of 2006, and the Profiles survey was administered in spring of that year. Thus, some Oregon schools may have already responded to the federal requirement. If this was the case, our analysis of the Washington trends may have underestimated the real effect of the Washington mandate. Finally, this study describes the policy environment in the Pacific Northwest, which may differ in other regions.

Future studies should examine the relationship between state and federal laws and the quality of PAN policy implementation in schools and the association of PAN policy with youth PAN outcomes.

### Conclusion

Our results suggest that a statewide mandate had a modest positive effect on PAN policies and practices in schools. Government policy is potentially an effective tool for addressing the childhood obesity epidemic through improvements in PAN environments in schools.

## Figures and Tables

**Table 1:** Characteristics, Middle and High Schools, Washington State and Oregon, School Health Profiles Survey, 2002, 2004, and 2006

Year	Washington, n (%)			Oregon, n (%)		

	2002	2004	2006	2002	2004	2006
Total middle and high schools	1,542	1,525	1,489	832	920	1,038
Participated in survey^a^	296 (19.2)	537 (35.2)	599 (40.2)	192 (23.1)	262 (28.5)	277 (26.7)
Did not participate in survey	1,246 (80.8)	988 (64.8)	890 (59.8)	640 (76.9)	658 (71.5)	761 (73.3)
Excluded schools^b^^,^ c	57 (19.3)	86 (16.0)	92 (15.4)	29 (15.1)	46 (17.6)	50 (18.1)
Study sample^b^	239 (15.4)	451 (84.0)	507 (84.6)	163 (84.9)	216 (82.4)	227 (81.9)
Middle schools in study sample	120 (7.7)	224 (14.6)	260 (17.6)	86 (10.3)	119 (12.9)	127 (12.2)
High schools in study sample^d^	119 (7.7)	227 (14.8)	247 (16.5)	77 (9.2)	97 (10.5)	100 (9.6)

Abbreviations: CI, confidence interval; PA, physical activity; PE, physical education.

a Percentage of all schools.

b Percentage of all schools participating in School Health Profiles survey.

c Schools excluded from the study were alternative schools, combined middle and high schools, and schools with an enrollment of fewer than 15 students per grade or no standardized health and physical education curriculum.

d Percentage of schools in study sample.

**Table 2:** Demographics of Schools Participating in the School Health Profiles Survey in Washington State and Oregon, 2002–2006^a^

Characteristic^b^	Washington n (%)(n = 1,197)	Oregon n (%)(n = 606)
High socioeconomic status^c^	561 (46.9)	220 (36.3)
Urban area^d^	796 (66.5)	319 (52.6)
High score^e^	631 (52.7)	385 (63.5)

Abbreviations: CI, confidence interval; PA, physical activity; PE, physical education.

a Data are from Washington State Department of Education (http://reportcard.ospi.k12.wa.us/summary.aspx?groupLevel=District&year=2010-11) and Oregon Department of Education (http://www.ode.state.or.us/data/reports/toc.aspx#General%20ODE%20Report) and include schools with enrollment of at least 15 students per grade and a standardized health and physical education curriculum; excludes alternative schools, combined middle/high schools, and schools with other or unknown grade combinations. Some schools are represented in more than 1 year.

b Averaged over the 2002, 2004, and 2006  study years.

c Schools with fewer than one-third of students enrolled in the free and reduced-price lunch program.

d Schools in urban areas defined by Rural Area Commuting Area codes (13).

e Schools with more than half of students meeting state standards on statewide achievement tests.

**Table 3:** Prevalence of Washington State Middle and High Schools With Physical Activity and Nutrition Policy Measures in 2002, 2004, and 2006 and Estimated Annual Trend

Policy Outcome Measure	2002 Mean, % (95% CI)	2004 Mean, % (95% CI)	2006 Mean, % (95% CI)	Estimated Annual Trend^a^, (95% CI)	*P* Value^b^
**Nutrition**					
**Food-choice education**					
Middle school	75.4 (65.3-85.6)	79.9 (72.8-87.1)	88.5 (84.0-93.0)	1.6 (1.6 to 1.6)	.10
High school	87.1 (79.8-94.5)	89.1 (83.9-94.2)	92.5 (89.2-95.8)		
**Healthy-weight education**					
Middle school	77.3 (67.1-87.5)	88.1 (82.6-93.5)	89.7 (85.6-93.8)	1.0 (1.0 to 1.1)	.13
High school	89.2 (82.9-95.5)	91.4 (86.7-96.2)	92.7 (89.2-96.2)		
**General nutrition education**					
Middle school	73.6 (64.5-82.7)	79.3 (73.1-85.6)	82.5 (77.8-87.1)	0.4 (0.4 to 0.5)	.37
High school	86.3 (80.0-92.5)	86.5 (81.7-91.4)	86.6 (83.0-90.2)		
**Restricted access competitive foods (type of food)**					
Middle school	31.6 (24.7-38.5)	35.4 (29.3-41.5)	63.6 (58.9-68.4)	3.2 (3.2 to 3.2)	<.001
High school	14.9 (8.6-21.3)	8.8 (5.1-12.5)	37.8 (32.4-43.3)		
**Restricted access competitive food (time of day)**					
Middle school	41.6 (35.0-48.2)	49.5 (43.8-55.2)	68.3 (63.8-72.7)	3.9(3.9 to 4.0)	<.001
High school	23.1 (17.4-28.8)	17.9 (13.3-22.4)	48.0 (42.6-53.3)		
**Healthy food options**					
Middle school	66.0 (59.9-72.0)	73.0 (68.3-77.6)	64.1 (60.6-67.7)	−0.2 (−0.3 to −0.2)	.03
High school	78.3 (73.4-83.3)	76.5 (72.2-77.9)	75.0 (72.1-77.9)		
**School food practices**					
Middle school	9.0 (5.3-12.6)	10.6 (6.9-14.3)	22.2 (18.6-25.9)	0.3 (0.3 to 0.4)	.55
High school	17.7 (11.7-23.7)	11.6 (7.7-15.5)	18.6 (15.0-22.1)		
**Physical activity**					
**Life fitness knowledge**					
Middle school	69.6 (60.2-79.1)	79.8 (73.8-85.8)	75.9 (70.5-81.3)	0.4 (0.4 to 0.4)	.59
High school	77.9 (70.7-85.1)	78.5 (72.5-84.5)	80.5 (75.8-85.3)		
**Skills knowledge**					
Middle school	60.1 (49.5-70.7)	74.2 (66.3-82.0)	68.5 (61.8-75.2)	−0.1 (−0.1 to −0.1)	.35
High school	71.5 (62.4-80.6)	73.1 (65.6-80.7)	73.8 (68.0-79.6)		
**Safety knowledge**					
Middle school	64.9 (55.3-74.5)	75.1 (67.8-82.4)	73.8 (67.8-79.8)	−0.2 (−0.2 to −0.2)	.62
High school	75.8 (67.7-83.8)	77.7 (71.1-84.4)	76.2 (70.6-81.7)		
**Facilitators for PA**					
Middle school	77.4 (72.4-82.3)	71.8 (66.9-76.7)	70.5 (67.3-73.7)	−0.7 (−0.7 to −0.7)	.06
High school	56.7 (50.7-62.8)	53.7 (48.3-59.1)	50.3 (46.9-53.7)		
**No PE exemptions for sports**					
Middle school	76.3 (69.0-83.6)	74.8 (68.3-81.3)	87.4 (84.3-90.5)	−0.4 (−0.4 to −0.4)	.38
High school	58.7 (50.6-66.8)	61.8 (55.1-68.5)	70.7 (66.6-74.8)		
**No PE exemptions for community activities**					
Middle school	84.2 (76.8-91.6)	91.0 (86.2-95.9)	91.5 (88.8-94.1)	−1.0 (-1.0 to −0.9)	.15
High school	69.6 (60.1-79.0)	58.0 (49.1-66.9)	81.3 (77.3-85.3)		
**No PE exemptions for academics**					
Middle school	75.8 (67.3-84.2)	71.5 (63.8-79.3)	82.0 (78.4-85.6)	−1.1 (−1.1 to −1.1)	.17
High school	69.6 (60.1-79.0)	63.3 (54.7-72.0)	73.2 (68.7-77.7)		
**PE teacher certification**					
Middle school	90.5 (84.8-96.1)	87.9 (82.3-93.5)	84.5 (79.9-89.2)	−1.5 (−1.5 to −1.5)	.01
High school	91.7 (86.1-97.2)	92.7 (88.2-97.3)	94.4 (91.3-97.5)		
**PE completion requirement**					
Middle school	16.8 (9.5-24.2)	13.7 (7.8-19.7)	12.9 (8.5-17.3)	−0.8 (−0.8 to −0.7)	.18
High school	92.3 (86.8-97.8)	91.9 (87.0-96.7)	90.9 (87.0-94.8)		
**PE requirement**					
Middle school	100.0 (100.0-100.0)	97.8 (95.3-100.3)	99.6 (98.7-100.4)	−0.2 (−0.2 to −0.2)	.48
High school	94.8 (90.4-99.3)	96.8 (93.7-99.9)	96.8 (94.5-99.1)		

Abbreviations: CI, confidence interval; PA, physical activity; PE, physical education.

a Estimated annual trend based on Washington and Oregon data as percentage point increase or decrease in absence of 2005 Washington law, middle and high schools combined.

b *P* values from logit model indicate the significance of the increase or decrease in the annual trend.

**Table 4:** Effect of Washington State Physical Activity and Nutrition Mandate on School Nutrition Policies and Practices, 2006

Policy Outcome Measure	Percentage Point Change in Schools With Policy (95% CI)^a^	*P *Value^b^	Strata Contrast: *P* Value, Comparison of Middle Schools to High Schools^c^
**Food-choice education**			
Middle school	0.7 (−7.2 to 8.6)	.62	.88
High school	0.3 (−5.9 to 6.4)	.64	
**Healthy-weight education**			
Middle school	−1.3 (−8.7 to 6.1)	.81	.89
High school	-4.0 (−9.7 to 1.6)	.64	
**General nutrition education**			
Middle school	−1.4 (−9.0 to 6.2)	.80	.46
High school	−4.3 (−11.0 to 2.4)	.64	
**Restricted access competitive foods (type of food)**			
Middle school	20.0 (11.5 to 28.5)	<.001	.44
High school	18.8 (10.4 to 27.2)	<.001	
**Restricted access competitive food (time of day)**			
Middle school	10.7 (2.5 to 18.9)	.06	.03
High school	19.2 (11.1 to 27.3)	<.001	
**Healthy food options**			
Middle school	−5.9 (−12.3 to 0.5)	.002	.27
High school	−2.0 (−7.6 to 3.5)	<.001	
**School food practices**			
Middle school	10.4 (4.4 to 16.4)	.001	.06
High school	4.6 (−1.5 to 10.6)	.14	

Abbreviation: CI, confidence interval.

a Effect of the statewide law on selected physical activity and nutrition policies and practices in percentage point terms as measured by the magnitude of the difference from the expected trend in absence of the law, taking into account the trend in Oregon.

b Significance of the difference from the expected trend.

c *P* values from logit model

**Table 5:** Effect of Washington State Physical Activity and Nutrition Mandate on School Physical Activity Policies and Practices, 2006

Policy Outcome Measure	Percentage Point Change in Schools With Policy (95% CI)^a^	*P* Value^b^	Strata Contrast: *P* Value, Comparison of Middle Schools to High Schools^c^
**Life fitness knowledge**			
Middle school	−3.8 (−12.4 to 4.0)	.97	.31
High school	−0.6 (−7.9 to 6.7)	.32	
**Skills knowledge**			
Middle School	−0.4 (−10.8 to 10.0)	.33	.73
High school	−1.6 (−10.6 to 7.4)	.48	
**Safety knowledge**			
Middle school	0.6 (−9.2 to 10.4)	.68	.88
High school	−1.3 (−9.5 to 6.9)	.53	
**Facilitators for PA**			
Middle school	−1.1 (−7.0 to 4.8)	.08	.29
High school	−5.9 (−12.0 to 0.2)	.43	
**No PE exemptions for sports**			
Middle school	16.6 (9.5 to 23.7)	<.001	.004
High school	8.7 (2.2 to 15.2)	.24	
**No PE exemptions for community activities**			
Middle school	12.8 (6.1 to 19.5)	<.001	.63
High school	14.4 (6.8 to 22.0)	<.001	
**No PE exemptions for academics**			
Middle school	18.1 (9.7 to 26.5)	<.001	.02
High School	9.0 (−0.2 to 18.2)	.06	
PE teacher certification			
Middle school	−2.3 (−9.9 to 5.3)	.56	.16
High school	2.3 (−2.7 to 7.1)	.39	
**PE completion requirement**			
Middle school	−5.3 (−12.2 to 1.6)	.13	.10
High school	0.8 (−6.5 to 8.1)	.84	
**PE requirement**			
Middle school	1.7 (−0.3 to 3.7)	.09	.01
High school	−1.8 (−4.2 to 0.6)	.15	

a Effect of the statewide law on selected physical activity and nutrition policies and practices in percentage terms as measured by the magnitude of the difference from the expected trend in absence of the law.

b Significance of the difference from the expected trend.

c *P* values from logit model; test used

## Appendices

### Table 1. Nutrition Policy Outcome Measures: Nutrition Factors Generated From Factor Analysis and Component Variables From Individual Questions in School Health Profiles, 2002-2006

**Table:**

Policy Outcome Measure: Factor	Component Variables From Individual Questions in Profiles
Food-choice education	Teach to choose foods that are low in fat, saturated fat, and cholesterol
	Teach to eat more fruits, vegetables, and grains
	Teach to use sugars in moderation
	Teach to use salt and sodium in moderation
	Teach to eat more calcium-rich food

Healthy-weight education	Teach the risks of unhealthy weight control practices
	Teach to accept body size differences
	Teach to balance food intake and physical activity
	Teach about eating disorders

General nutrition education	Teach food guidance using MyPyramid
	Teach benefits of healthy eating
	Teach to prepare healthy meals and snacks
	Teach food safety
	Teach use of food labels

Restricted access to competitive foods (type of food)	Report not able to purchase chocolate candy from school vending machines or store
	Report not able to purchase other kinds of candy from school vending machines or store
	Report not able to purchase salty snacks not low in fat (eg, potato chips from school vending machines or store
	Report not able to purchase soft drinks or fruit drinks that are not 100% juice from school vending machines or store

Restricted access to competitive foods (time of day)	Report not able to purchase snack food or beverages before classes in the morning
	Report not able to purchase snack food or beverages during lunch period
	Report not able to purchase snack food or beverages during any school hours when meals are not served

Healthy food options	Report able to purchase 1% or skim milk from school vending machines or store
	Report able to purchase 2% or whole milk (plain or flavored)
	Report able to purchase low-fat cookies, crackers, cakes, pastries, baked goods from school vending machines or store
	Report able to purchase low-fat salty snacks from (eg, pretzels, baked chips) from school vending machines or store
	Report able to purchase fruit or vegetables from school vending machines or store
	Report able to purchase bottled water from school vending machines or store
	Report able to purchase sports drinks from school vending machines or store

School food practices	Report students usually have 20 or more minutes to eat lunch once seated
	Report school/district policy that fruits/vegetables served at parties, after-school programs, staff/parent meetings, concessions, etc.

### Table 2. Physical Activity Policy Outcome Measures: Physical Activity Factors Generated From Factor Analysis and Component Variables From Individual Questions in School Health Profiles, 2002-2006

**Table:**

Policy Outcome Measures: Factor	Component Variables From Individual Questions in Profiles
Life fitness knowledge	Teach difference between PA, exercise, and fitness
	Teach overcoming barriers to PA
	Teach decreasing sedentary activities such as watching television
	Teach opportunities for physical activities in the community
	Teach health-related fitness (cardio, endurance, strength, flexibility, composition)
	Teach the physical, psychological, or social benefits of PA

Skills knowledge	Teach how much PA is enough (frequency, intensity, time, type)
	Teach phases of a workout (warm-up, workout, cool-down)
	Teach monitoring progress to reach individual PA plan goals
	Teach developing an individualized PA plan

Safety knowledge	Teach preventing injury during PA
	Teach weather-related safety (heat stroke, hypothermia, sunburn)
	Teach dangers of using performance-enhancing drugs such as steroids

Facilitators for PA	Provide community sponsored programs at school outside school hours
	Promote walking/biking to and from school (activities, safe/preferable routes, storage)
	Provide transportation home after-school intramural activity or PA clubs

No PE exemptions for sports	No exemption from PE because of student participation in school sports
	No exemption from PE because of student participation in community sports
	No exemption from PE because of students' high physical fitness competence score

No PE exemptions for community activities	No exemption from PE because of student participation in community service
	No exemption from PE because of student participation in school activities (eg, ROTC)

No PE exemptions for academics	No exemption from PE because of student participation in vocational training
	No exemption from PE because of student participation in other courses (eg, math, science)

PE teacher certification	Requirement for newly hired PE teachers to be certified, licensed, or endorsed by the state

PE completion requirement	Requirement for students to repeat physical education if course is failed

PE requirement	Requirement for physical education for students in grades 6 through 12

Abbreviations: PA, physical activity; PE, physical education; ROTC, Reserve Office Training Corps.
